# Synergistic anticancer effect of cisplatin and Chal-24 combination through IAP and c-FLIP_L_ degradation, Ripoptosome formation and autophagy-mediated apoptosis

**DOI:** 10.18632/oncotarget.2746

**Published:** 2015-02-12

**Authors:** Shaoqing Shi, Qiong Wang, Jennings Xu, Jun-Ho Jang, Mabel T. Padilla, Toru Nyunoya, Chengguo Xing, Lin Zhang, Yong Lin

**Affiliations:** ^1^ Department of Immunology, College of Basic and Forensic Medicine, Sichuan University, Chengdu, China; ^2^ Molecular Biology and Lung Cancer Program, Lovelace Respiratory Research Institute, Albuquerque, NM, USA; ^3^ Laboratory of Molecular and Translational Medicine, West China Second University Hospital, Sichuan University, Chengdu, China; ^4^ Division of Pulmonary and Critical Care Medicine, University of New Mexico and New Mexico VA Health Care System, Albuquerque, NM, USA; ^5^ Department of Medicinal Chemistry, University of Minnesota, Minneapolis, MN, USA

**Keywords:** autophagy, apoptosis, c-IAP, c-FLIP, cisplatin, Chal-24

## Abstract

Drug resistance is a major hurdle in anticancer chemotherapy. Combined therapy using drugs with distinct mechanisms of function may increase anticancer efficacy. We have recently identified the novel chalcone derivative, chalcone-24 (Chal-24), as a potential therapeutic that kills cancer cells through activation of an autophagy-mediated necroptosis pathway. In this report, we investigated if Chal-24 can be combined with the frontline genotoxic anticancer drug, cisplatin for cancer therapy. The combination of Chal-24 and cisplatin synergistically induced apoptotic cytotoxicity in lung cancer cell lines, which was dependent on Chal-24-induced autophagy. While cisplatin slightly potentiated the JNK/Bcl2/Beclin1 pathway for autophagy activation, its combination with Chal-24 strongly triggered proteasomal degradation of the cellular inhibitor of apoptosis proteins (c-IAPs) and formation of the Ripoptosome complex that contains RIP1, FADD and caspase 8. Furthermore, the cisplatin and Chal-24 combination induced dramatic degradation of cellular FLICE (FADD-like IL-1β-converting enzyme)-inhibitory protein large (cFLIP_L_) which suppresses Ripoptosome-mediated apoptosis activation. These results establish a novel mechanism for potentiation of anticancer activity with the combination of Chal-24 and cisplatin: to enhance apoptosis signaling through Ripoptosome formation and to release the apoptosis brake through c-FLIP_L_ degradation. Altogether, our work suggests that the combination of Chal-24 and cisplatin could be employed to improve chemotherapy efficacy.

## INTRODUCTION

Because chemoresistance is the major hurdle in anticancer therapy, sensitizing cancer cells to chemotherapy is the main challenge to improve survival in cancer patients. The main mechanism of current anticancer therapeutics is to directly kill cancer cells [1]. However, primary or acquired chemoresistance in cancer cells blunts the efficacy of anticancer drugs, thereby causing therapy failure [2, 3]. The main mechanism by which chemotherapeutics kill cancer cells is through activation of apoptosis and thus, evasion of apoptosis substantially contributes to chemoresistance [4–6]. Therefore, releasing the apoptosis brake in cancer cells will increase cytotoxicity induced by anticancer chemotherapy [5]. Combination therapy that combines drugs with distinct mechanisms of function, would significantly increase anticancer efficacy.

Two major apoptosis activation pathways exist. The extrinsic pathway is activated through the ligation of tumor necrosis factor (TNF) family cytokines to their respective receptors initiating formation of the death- inducing signaling complex (DISC) [7, 8]. Intracellular stresses activate the intrinsic pathway that involves loss of mitochondrial potential and release of mitochondrial proteins such as cytochrome C and SMAC to form the apoptosome consisting of cytochrome C, APAF-1 and caspase 9. Many anticancer drugs, such as cisplatin, activate the intrinsic apoptosis pathway through DNA damage and genotoxic stress [9, 10]. Recently, it was found that genotoxic anticancer therapeutics such as etoposide induce an alternative apoptosis pathway that involves formation of a complex called the Ripoptosome, consisting of RIP1, FADD, and caspase 8 [11, 12]. This pathway is likely activated through degradation of the cellular inhibitor of apoptosis proteins (c-IAPs) [11, 12]. The activation of caspase 8 in the Ripoptosome is negatively regulated by cellular FLICE (FADD-like IL-1β-converting enzyme)-inhibitory protein large (cFLIP_L_), and thus, suppressing cFLIP_L_ would promote Ripoptosome-mediated apoptosis [11, 13]. Additionally, autophagy, a catabolic process for degradation and recycling of long-lived proteins and organelles, can also lead to apoptotic cell death [14, 15]. Thus, these apoptosis activating pathways could be exploited for chemosensitization.

Our recent work has suggested the novel chalcone derivative, Chal-24, as a potential anticancer agent [16, 17]. Without obvious signs of toxicity in nude mice, Chal-24 potently inhibited xenografted human tumor growth [16]. At relatively high concentrations (> 4 μM), Chal-24 activates autophagy through JNK-mediated phosphorylation of Bcl-2 and Bcl-xL, leading to disruption of the Beclin1/Bcl-2 and Beclin1/Bcl-xL complexes, with subsequent degradation of the c-IAP proteins and formation of the Ripoptosome complex, resulting in necroptosis in cancer cells [17]. However, at lower concentrations (≤ 1 μM) Chal-24 mainly induces apoptosis and cell cycle arrest [17, 18]. The potential use of Chal-24 in combination with other anticancer drugs in chemotherapy has not been explored.

In this study, we hypothesized that due to their distinct cytotoxic mechanisms, Chal-24 and cisplatin would synergistically kill cancer cells. We found that the combination of Chal-24 and cisplatin synergistically increased cytotoxicity, which was associated with autophagy-mediated apoptosis. While cisplatin slightly potentiated autophagy activation, its combination with Chal-24 strongly enhanced proteasomal degradation of c-IAPs and formation of the Ripoptosome complex. Furthermore, the cisplatin and Chal-24 combination induced dramatic degradation of the Ripoptosome-inhibiting factor, c-FLIPL. Our results establish a novel mechanism for potentiation of anticancer activity with the combination of Chal-24 and cisplatin: to enhance apoptosis signaling through Ripoptosome formation and to release the apoptosis brake through c-FLIPL degradation. Data from this study suggest that the combination of Chal-24 and cisplatin could be employed to improve chemotherapy efficacy.

## RESULTS

### Combination of Chal-24 and cisplatin results in synergistic apoptotic cell death in lung cancer cells

**Figure 1:**
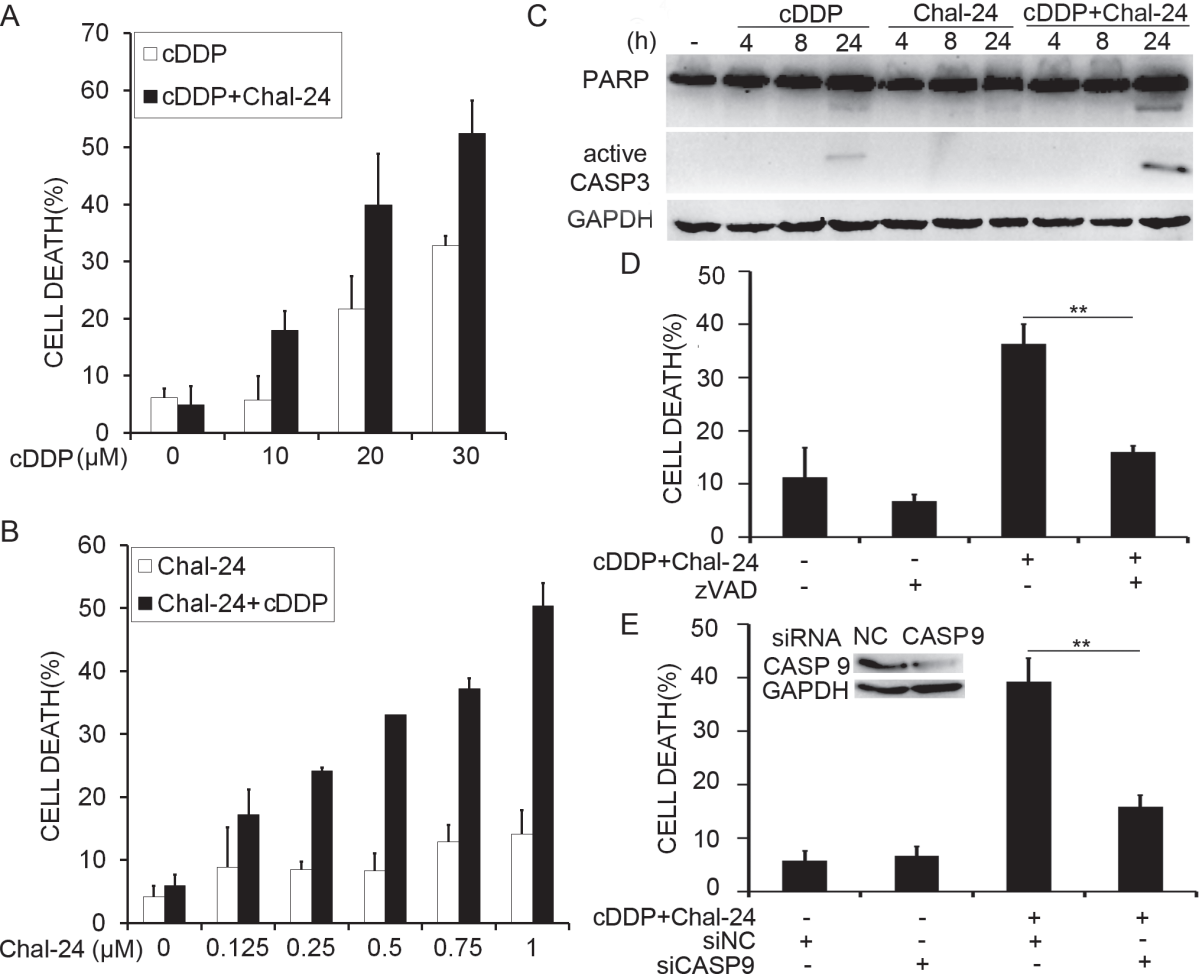
Combination of Chal-24 and cisplatin results in synergistic apoptotic cell death in lung cancer cells **(A)** A549 cells were treated with cisplatin (10–30 μM) and Chal-24 (0.5 μM) for 48 h. Cell death were measured by LDH release assay. Data shown are mean ± SD, representative of three independent experiments. **(B)** A549 cells were treated with cisplatin (10 μM) and Chal-24 (0.125–1 μM) for 48 h. Cell death were measured as described in A. **(C)** A549 cells were treated with cisplatin (10 μM) and Chal-24 (1 μM) for indicated time periods. Cell extracts were resolved in 12% SDS-PAGE gels. PARP and active caspase-3 were detected by Western blot. GAPDH was detected as an input control. **(D)** A549 cells were pretreated with z-VAD (10 μM) for 30 min, followed by 48 h treatment with cisplatin (10 μM) and Chal-24 (1 μM), cell death were detected as described in A. **(E)** the cells were transfected with indicated siRNA for 24 h, and treated with (10 μM) and Chal-24 (1 μM) for additional 48 h. Cell death was measured as described in A. **p < 0.01. Insert, knockdown of caspase 9 expression was confirmed by Western blot.

To investigate the potential of combination of Chal-24 and cisplatin for chemotherapy, we first treated A549 cells with increasing concentrations of cisplatin (10–30 μM) and a fixed concentration of Chal-24 (0.5 μM). Cell death was detected and quantified by LDH assay. While treatment with Chal-24 alone at this low concentration caused little cell death, Chal-24 synergistically potentiated cisplatin-induced cytotoxicity in a cisplatin dose-dependent manner (Combination index, CI: 0.5625) (Fig. 1A). Consistently, a similar synergy was also observed with increasing concentrations of Chal-24 (0.125–1.0 μM) and a fixed cisplatin dose (10 μM) (CI: 0.375) (Fig. 1B). Because Chal-24 at 1 μM exerted limited cytotoxicity but effectively potentiated cisplatin-induced cell death, this concentration of Chal-24 was used for later experiments. To confirm whether the potentiation of cytotoxicity induced by cisplatin and Chal-24 combination is a common phenomenon, three additional human lung cancer cell lines, H23, H460 and H1299, were tested under similar conditions. Comparable sensitization in the anticancer effect was seen in all these cell lines (Figs. S1A–S1C). Collectively, these results suggest that Chal-24 and cisplatin synergistically kill lung cancer cells.

Next, we performed a series of experiments to elucidate the mechanism of cell death induced by cisplatin and Chal-24 combination. Results from all these experiments supported that apoptosis was the main cell death mode: with activation of caspase 3 and cleavage of PARP (hallmarks of apoptosis) (Figs. 1C, S1D); with morphological apoptotic features in dead cells detected by acridine orange/ethidium bromide (AO/EB) staining (cell shrinkage, cell membrane blebbing, and nuclear condensation) (data not shown); Annexin V positive in the main portion of dead cells (data not shown); and with effective suppression of cell death by the pan-caspase inhibitor z-VAD or caspase 9 knockdown (Figs. 1D, 1E, S1E and S1F). These results suggest that the cisplatin and Chal-24 combination mainly induces apoptotic cytotoxicity in cancer cells.

### Autophagy is required for cell death induced by the Chal-24 and cisplatin combination

**Figure 2:**
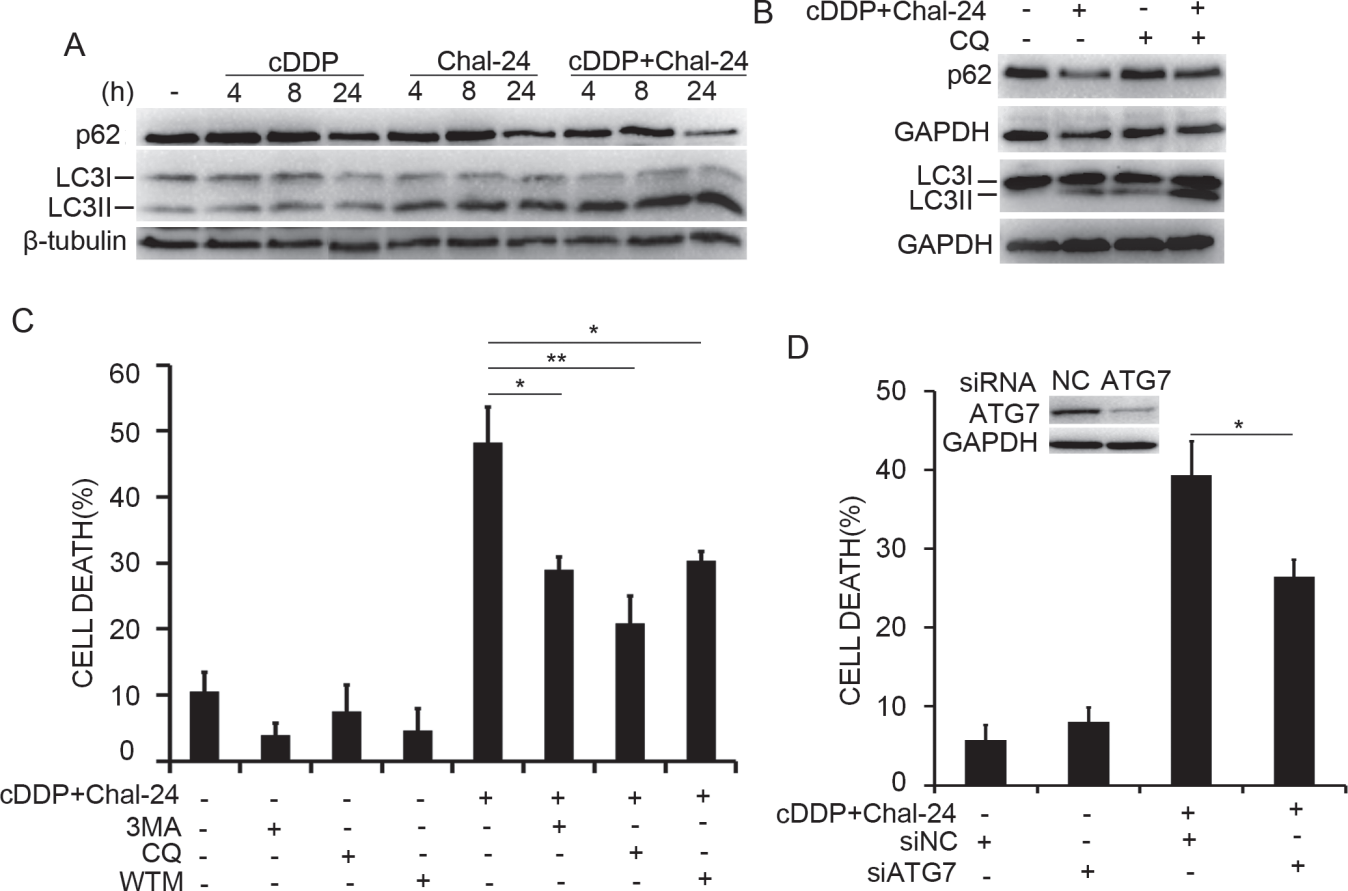
Autophagy is required for cell death induced by Chal-24 and cisplatin combination **(A)** A549 cells were treated with cisplatin (10 μM), Chal-24 (1 μM) alone or in combination for the indicated times. The indicated proteins were detected by Western blot. β-tubulin was used as an input control. **(B)** the cells were preteated with chloroquine (CQ, 20 μM) for 30 min, and then treated with cisplatin and Chal-24 (24 h or 4 h for upper panel for p62 detection and lower panel for LC3 detection, respectively). The indicated proteins were detected by Western blot. GAPDH was detected as an input control. **(C)** A549 cells were pretreated with autophagy inhibitors (CQ, 20 μM; WTM, 1 μM; 3MA, 10 μM) for 30 min, followed by cisplatin (10 μM) and Chal-24 (1 μM) co-treatment for an additional 48 h, cell death was measured as described in Figure 1. **(D)** the cells were transfected with the indicated siRNA for 24 h, then the cells were treated with cisplatin (10 μM) and Chal-24 (1 μM) for 48 h, cell death was measured by LDH assay. *p < 0.05, **p < 0.01. Insert, knockdown of ATG7 expression was confirmed by Western blot.

We previously found that Chal-24 induces autophagy-dependent necroptosis at higher concentrations (8–32 μM) [17]. Therefore, we investigated whether the combination of cisplatin and a lower concentration of Chal-24 induces autophagy. Chal-24 at 1 μM evidently induced autophagy, which was shown as conversion of LC3I to LC3II (4–24 h) and reduction of p62 (24 h). While cisplatin alone had little effect on the autophagy markers, the co-treatment with cisplatin and Chal-24 slightly potentiated autophagy (Fig. 2A). Induction of autophagy by the cisplatin and Chal-24 combination was further confirmed by an autophagic flux assay (Fig. 2B). The role of autophagy in cell death induced by the Chal-24 and cisplatin combination was examined with the use of different autophagy inhibitors to inhibit different steps in the autophagy process. All these three inhibitors significantly suppressed cytotoxicity induced by Chal-24 and cisplatin co-treatment (Figs. 2C and S2). Supporting this observation, knockdown of the key autophagy mediator, ATG7, effectively attenuated the cytotoxicity induced by cisplatin plus Chal-24 (Fig. 2D). These data suggest that the cisplatin and Chal-24 combination induces cytotoxicity depending on Chal-24-induced autophagy.

### Combination of cisplatin and Chal-24 activates the JNK-mediated autophagy pathway involving phosphorylation of Bcl-2

**Figure 3:**
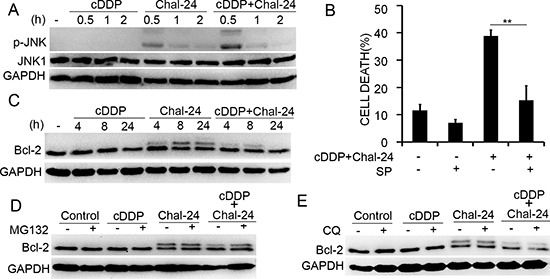
Combination of cisplatin and Chal-24 activates JNK-and phosphorylation of Bcl-2 **(A)** A549 cells were treated with cisplatin (10 μM) and Chal-24 (1 μM) alone or in combination for indicated times. JNK1 and phospho-JNK were examined by Western blot. GAPDH was detected as an input control. **(B)** the cells were pretreated with SP600125 (10 μM) for 30 min, and then treated with cisplatin (10 μM) and Chal-24 (1 μM) for an additional 48 h, cell death were detected by LDH assay. **p < 0.01. **(C)** A549 cells were treated with cisplatin (10 μM) and Chal-24 (1 μM) alone or in combination for indicated times. The indicated proteins were detected by Western blot. GAPDH was used as an input control. **(D, E)** A549 cells were pretreated with CQ (20 μM) or MG132 (5 μM) for 30 min, respectively, then treated with cisplatin (10 μM), Chal-24 (1 μM) or in combination for an additional 8 h. The indicated proteins were detected by Western blot, GAPDH was detected as an input control.

Our recent studies showed that Chal-24 induces autophagy through activation of JNK, phosphorylation of Bcl-2, and disruption of the Beclin1/Bcl-2 complex [17]. Therefore, we investigated whether the cisplatin and Chal-24 combination induces autophagy by the same mechanism. While cisplatin barely affected JNK activity, the drug combination induced a higher level of JNK activation compared to that of Chal-24 alone (Figs. 3A and S3A). Inhibition of JNK effectively suppressed the cytotoxicity induced by cisplatin and Chal-24 co-treatment in both A549 and H460 cell lines (Figs. 3B, S3B). Moreover, Chal-24 induced phosphorylation of Bcl-2 (Figs. 3C, and S3C). Interestingly, the co-treatment with cisplatin resulted in reduction of phosphorylated Bcl-2 (Figs. 3C, 3D, 3E). This was likely due to degradation of this phosphorylated protein in proteasome, because phosphorylated Bcl-2 was restored by the proteasome inhibitor MG132 but not the lysosome inhibitor chloroquine (Figs. 3D, 3E). Together with previous studies showing that JNK-mediated phosphorylated Bcl-2 releases Beclin-1 to activate autophagy [17], these results suggest that the cisplatin and Chal-24 combination induces cell death dependent on autophagy activation involving JNK-mediated phosphorylation and degradation of Bcl-2. Furthermore, suppression of JNK inhibited autophagy, caspase 3 activation and PARP cleavage induced by combination of Chal-24 and cisplatin (Fig. S1F and data not shown), suggesting JNK is involved in autophagy-mediated apoptosis induced by this drug combination.

### Combination of cisplatin and Chal-24 induces ERK-mediated degradation of the IAP proteins

**Figure 4:**
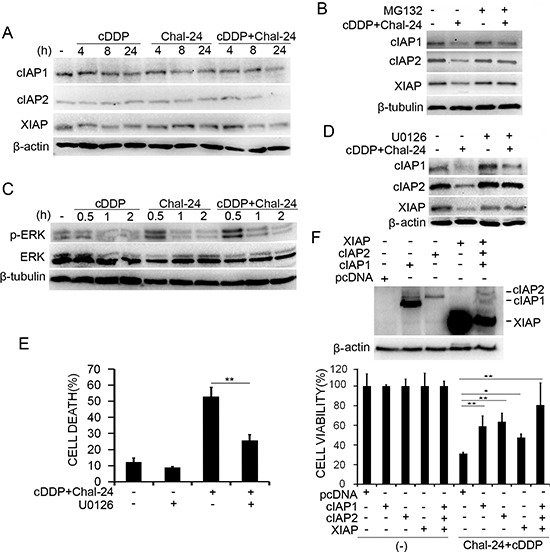
Combination of cisplatin and Chal-24 induces ERK-mediated degradation of the IAP proteins **(A)** A549 cells were treated with cisplatin (10 μM), Chal-24 (1 μM), or in combination for indicated times. The indicated proteins were detected by Western blot. β-actin was detected as an input control. **(B)** the cells were pretreated with MG132 (5 μM) for 30 min, and then treated with cisplatin (10 μM) and Chal-24 (1 μM) for an additional 24 h, the indicated proteins were detected by Western blot. β-tubulin was detected as an input control. **(C)**, A549 cells were treated with cisplatin (10 μM), Chal-24 (1 μM), or in combination for indicated times. The indicated proteins were examined by Western blot. β-tubulin was detected as an input control. **(D)** the cells were pretreated with U0126 (10 μM) for 30 min, and then treated with cisplatin (10 μM) and Chal-24 (1 μM) for an additional 24 h, the indicated proteins were examined by Western blot. β-actin was detected as an input control. **(E)** the cells were pretreated with U0126 (10 μM) for 30 min, and then treated with cisplatin (10 μM) and Chal-24 (1 μM) for an additional 48 h, cell death was measured by LDH assay. **(F)** A549 cells were transfected with plasmids expressing the IAPs or the empty vector pcDNA with EGFP, and treated with cisplatin (10 μM) and Chal-24 (1 μM) for 40 h. Survival of EGFP-positive cells was quantified by counting live cells with green fluorescence. Data shown are mean ± SD. **p < 0.01. Upper, expression of transfected proteins was confirmed by Western blot.

Our recent studies found that Chal-24 at high concentrations causes ERK- and autophagy-dependent degradation of IAPs [17]. Because the IAP family proteins are important antiapoptotic factors [19–22], we then examined if the cisplatin and Chal-24 co-treatment impacts the expression of IAPs. Under the experimental condition with moderate drug concentrations, cisplatin or Chal-24 alone had little effect on the expression of cIAP1, cIAP2 and XIAP. However, the combination of these two agents dramatically suppressed the expression of these proteins (Figs. 4A, S4A). The proteasome inhibitor, MG132, significantly restored the expression of IAPs, suggesting that the cisplatin and Chal-24 co-treatment enhanced proteasomal degradation of the IAPs (Figs. 4B and S4B). Consistent with previous studies [17], Chal-24 alone at a lower concentration also activated ERK, which was slightly increased by cisplatin (Fig. 4C and S4C). Inhibition of ERK significantly attenuated IAPs degradation and the cytotoxicity induced by cisplatin and Chal-24 co-treatment (Figs. 4D, 4E, S4D and S4E). In addition, cytotoxicity induced by cisplatin and Chal-24 co-treatment was substantially attenuated by ectopic expression of cIAP1, cIAP2 and XIAP (Fig. 4F). Furthermore, ERK suppression inhibited apoptosis but not autophagy (data not shown), consisting with that ERK is involved in IAP suppression but not autophagy [17]. Altogether, these results suggest that combined treatment with cisplatin and Chal-24 induces ERK-mediated proteasomal degradation of IAP proteins for promoting apoptosis.

### Combination of cisplatin and Chal-24 triggers Ripoptosome formation and cFLIP_L_ degradation

**Figure 5:**
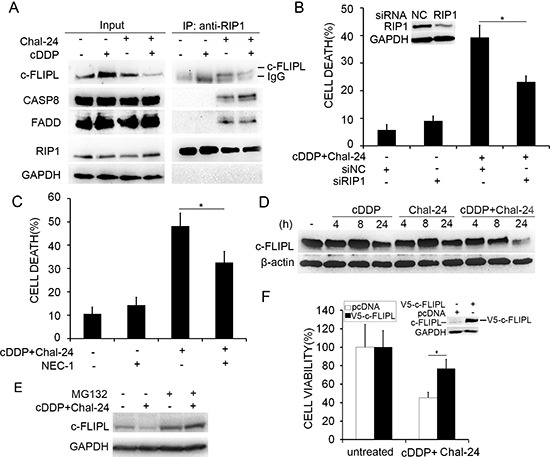
Combination of cisplatin and Chal-24 triggers Ripoptosome formation and c-FLIP_L_ degradation **(A)** A549 cells were treated with cisplatin (10 μM), Chal-24 (1 μM), or in combination for 24 h, the indicated proteins were detected by Western blot after co-immunoprecipitation with an anti-RIP1 antibody. **(B)** the cells were transfected with the indicated siRNA for 24 h, then the cells were treated with (10 μM) and Chal-24 (1 μM) for 48 h, cell death was measured by LDH assay. *p < 0.05. Inset, knockdown of RIP1 expression was confirmed by Western blot. **(C)** the cells were pretreated with necrostatin-1 (NEC-1, 10 μM) for 30 min, and then treated with cisplatin (10 μM) and Chal-24 (1 μM) for an additional 48 h, cell death was measured by LDH assay. *p < 0.05. D, A549 cells were treated with cisplatin (10 μM), Chal-24 (1 μM) alone, or in combination for indicated times. The indicated proteins were examined by Western blot. β-actin was detected as an input control. **(E)** the cells were pretreated with MG132 (5 μM) for 30 min, and then treated with cisplatin (10 μM) and Chal-24 (1 μM) for an additional 24 h. The indicated proteins were examined by Western blot. GAPDH was detected as an input control. **(F)** A549 cells were transfected with V5-c-FLIP_L_ or pcDNA with EGFP, and treated with cisplatin (10 μM) and Chal-24 (1 μM) for 40 h. Cell survival was quantified by counting cells with green fluorescence. Data shown are mean ± SD. *p < 0.05. Insert, expression of transfected protein was confirmed by Western blot.

**Figure 6:**
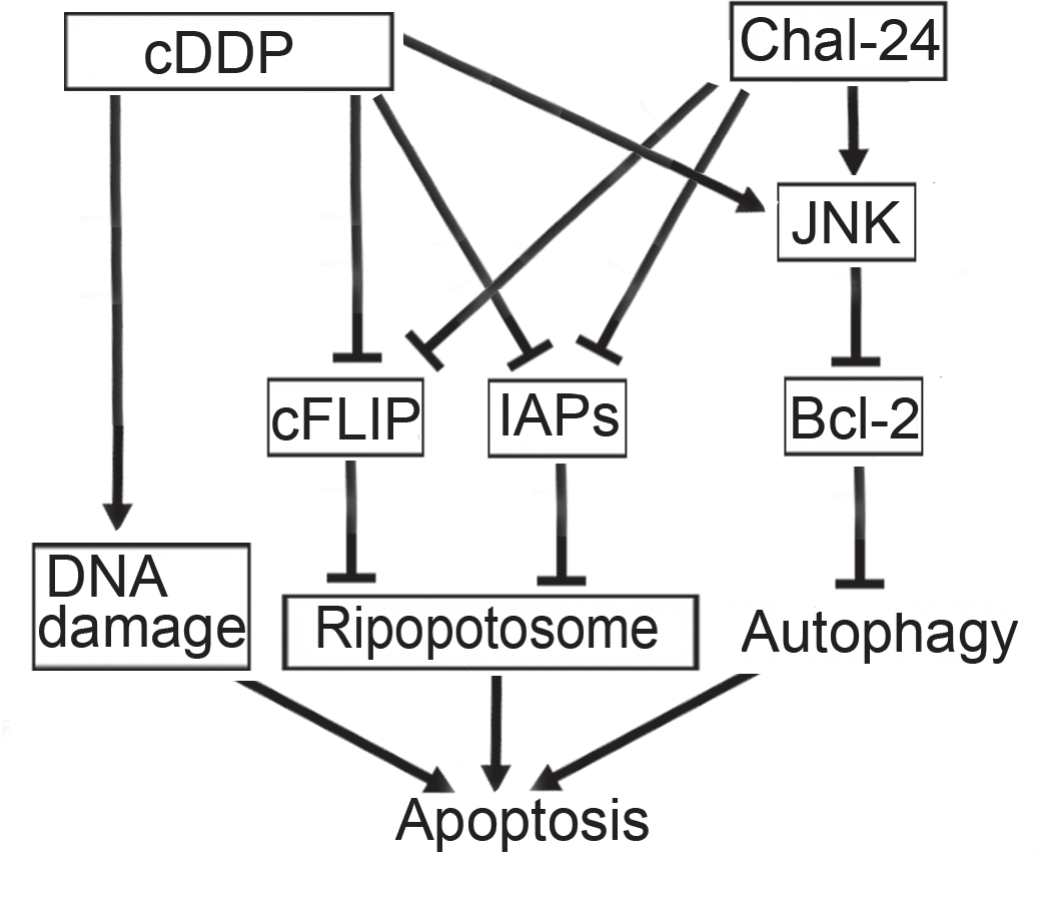
A model for Chal-24 and cisplatin combination in induction of apoptotic cell death Combination of Chal-24 and cisplatin kills cancer cells through apoptosis involving multiple pathways: the enhancement of Ripoptosome formation involving IAPs suppression and release the apoptosis brake involving c-FLIP degradation, autophagy involving JNK-mediated Bcl-2 phosphorylation, and cisplatin-induced DNA damage.

Because suppressing expression of cIAPs by genotoxic drugs or high concentrations of Chal-24 results in formation of the Ripoptosome complex consisting of RIP1, FADD, cFLIP_L_ and caspase 8 that mediates cell death [11–13, 17, 23], we examined, by co-immunoprecipitation with an anti-RIP1 antibody, if the cisplatin and Chal-24 co-treatment triggers Ripoptosome formation. Under this condition, Chal-24 but not cisplatin alone induced the formation of the Ripoptosome. Interestingly, the co-treatment with cisplatin enhanced the recruitment of caspase 8 to the Chal-24-induced Ripoptosome (Fig. 5A). RIP1 is the central factor for Ripoptosome formation and function [11, 13, 24, 25]. Knockdown of RIP1 expression with siRNA or suppression of RIP1 kinase activity with Necrostatin-1 significantly suppressed cytotoxicity induced by cisplatin and Chal-24 co-treatment (Fig. 5B, 5C, S5A), suggesting that the Ripoptosome is important for mediating the anticancer effect of cisplatin and Chal-24 co-treatment. cFLIP_L_ was reported to be a negative regulator of Ripoptosome-mediated apoptosis and cisplatin was shown to suppress cFLIP_L_ expression [11, 26, 27]. Thus, we investigated the effect of Chal-24 and cisplatin combination on cFLIP_L_ expression. In A549 cells, cisplatin individually caused a moderate decrease of cFLIP_L_ expression. However, the co-treatment with Chal-24 significantly promoted cisplatin-induced c-FLIP_L_ expression suppression (Fig. 5D). A similar trend for this effect was also detected in H460 cells, although cisplatin individually induced decrease of cFLIP_L_ is more evident at a later time point (24 h, Fig. S5B). The proteasome inhibitor MG132 significantly restored c-FLIP_L_ expression in cells treated with the Chal-24 and cisplatin combination (Fig. 5E), suggesting the co-treatment triggered proteasomal degradation of cFLIP_L_. Consistently, the recruitment of cFLIP_L_ to the Ripotosome was significantly reduced in the cisplatin and Chal-24 co-treated cells, which was associated with increased caspase 8 recruitment (Fig. 5A). Importantly, ectopic expression of cFLIP_L_ strongly attenuated cytotoxicity induced by cisplatin and Chal-24 co-treatment (Fig. 5F). Altogether, these data suggest that the combination of cisplatin and Chal-24 induces cell death by promoting Ripoptosome-mediated apoptosis (Fig. 6).

## DISCUSSION

In this study, we demonstrate that the combination of cisplatin and Chal-24 significantly potentiates apoptotic cytotoxicity in several human lung cancer cell lines. These results establish a novel mechanism for combination therapy with these agents that involves a series of processes: Chal-24 induces autophagy and Ripoptosome formation that are effectively enhanced by cisplatin, and cisplatin promotes degradation of c-FLIP_L_ to release the brake on Ripoptosome-mediated apoptosis. The autophagy- and Ripotosome-mediated apoptosis signaling pathways, together with the DNA damage-mediated apoptosis pathway activated by cisplatin, cooperatively contribute to the synergistic anticancer effect of cisplatin and Chal-24 combination (Fig. 6). This novel cancer cell killing mechanism could be exploited for chemotherapy, and the cisplatin and Chal-24 combination could be a potential effective approach for improving anticancer therapeutic efficacy and overcoming cancer chemoresistance.

Theoretically, combinations of drugs that cause a similar effect but function through distinct mechanisms could achieve a synergistic effect in outcome [28, 29]. It is well established that cisplatin kills cancer cells by crosslinking DNA resulting in DNA strand breaks during mitotic cell division to induce apoptosis [9, 10]. In contrast, Chal-24 kills cancer cells through autophagy- and Ripoptosome-mediated cell death [17]. Thus, we hypothesize the combination of these agents will synergistically kill cancer cells. Indeed, the results verified this hypothesis. In addition, acquired cisplatin resistance is common in patients receiving platinum-based therapy, which involves increased drug detoxification and DNA repair and apoptosis inhibition [30]. Chal-24 may prevent and attenuate acquired cisplatin resistance by targeting different pathways from those activated by cisplatin, which deserve further studies.

We reported recently that Chal-24 activates autophagy and Ripoptosome formation for cancer cell necroptosis at relatively high concentrations (> 8 μM) [17]. However, it was noticed that at a lower concentration (0.3 μM), Chal-24 induces apoptosis and cell cycle arrest [17, 18]. At the high Chal-24 concentrations, the JNK-mediated Bcl-2 and Bcl-xL phosphorylation pathway that disrupts the Beclin1/Bcl-2 and Belin1/Bcl-xL complexes is required for autophagy, and the ERK- and autophagy-mediated degradation of IAPs promotes Ripoptosome formation. Interestingly, we found in this report that these cancer cell-killing pathways can be activated by relatively low Chal-24 concentrations (≤ 1 μM) and further potentiated by cisplatin, although the cellular outcome is apoptosis instead of necroptosis. The outcome of apoptotic cell death is not surprising, because both the autophagy process and the Ripoptosome can lead to either apoptosis or necroptosis depending on cell context and the types of stimulation [12–14, 25, 31, 32]. Interestingly, mitosis inhibitors such as taxol induce Bcl-2 phosphorylation involving MAPKs such as JNK [33, 34]. However, consistent with literature [35], there is limited potentiation of cytotoxicity in Taxol and cisplatin co-treatment (data not shown). Thus, it is likely that the induction of JNK-mediated Bcl-2 phosphorylation is insufficient and additional important pathways are required for synergistic cytotoxicity induced by Chal-24 and cisplatin combination. It would be interesting to investigate in the future if Chal-24-induced and JNK-mediated Bcl-2 and Bcl-xL phosphorylation involves cell cycle signaling pathways.

The IAP proteins are important for promoting the NF-κB cell survival pathway and suppressing cell death during TNFR1 signaling [19, 36, 37]. IAPs are frequently overexpressed in cancer cells, which contributes to cell survival, chemoresistance, disease progression and poor prognosis [23]. Thus, IAPs are proposed to be targets for cancer chemotherapy [38]. One major IAP-regulated factor is RIP1, a key mediator of both cell survival and death pathways and the central protein of the Ripoptosome complex [23]. Under certain conditions such as DNA damage induced by DNA topoisomerase II inhibition, IAPs are degraded to trigger Ripoptosome formation, and the Ripoptosome serves as a cell death-inducing platform for either apoptosis or necroptosis [12, 13, 25]. The Ripoptosome-mediated cell death pathways are kept in check by c-FLIP_L_ [11, 12]. Data from the current study suggest that cisplatin is able to effectively cause FLIP_L_ degradation to release the brake on Ripoptosome-mediated cell death pathways. Interestingly, c-FLIP is overexpressed in cancer cells and involved in cancer pathogenesis [39]. It is also proposed that c-FLIP could be a target for cancer therapy [40]. Because the cisplatin and Chal-24 combination simultaneously suppresses both IAPs and c-FLIP, it could be an ideal approach to achieve synergistic anticancer efficacy by targeting these two antiapoptotic factors that function through different mechanisms. It is remarkable that p53 is a key factor for DNA damage response that affects the anticancer activity of cisplatin. Our results show that the response to cisplatin and Chal-24 combination in cells with p53 mutation (H23) or deletion (H1299) was comparable to cell lines having wild-type p53 (A549 and H460) (Figs. 1A and S1A–C). Furthermore, knockdown of p53 in A549 had no detectable effect on cell death induced by this drug combination (Data not shown). Thus, it is likely that the apoptotic response induced by combination of Chal-24 and cisplatin is independent of p53, making the combination useful in killing p53-mutanted cancer cells.

Altogether, our results establish a novel mechanism for killing cancer cells by the cisplatin and Chal-24 combination that involves induction of autophagy, formation of the Ripoptosome and degradation of IAPs and c-FLIP_L_ (Fig. 6), which may be exploited for improving chemotherapy efficacy. Further *in vivo* studies are warranted for determining the anticancer effectiveness and chemoresistance attenuation potential of this drug combination.

## MATERIALS AND METHODS

### Reagents

Cisplatin (479306) was from Sigma (St. Louis, MO). Anti-RIP1 (610458), JNK1 (544285), c-IAP2 (552782), FADD (556402), Caspase 8 (551242), Caspase 3 (559565) and p62 (610832) antibodies were from BD Biosciences (San Diego, CA, USA). Antibodies against Bcl-2 (sc-7382), and GAPDH (sc-32233) were from Santa Cruz Biotechnology (Santa Cruz, CA, USA). Anti-phospho-JNK (44682G) and phospho-ERK (AHO0061) were from Invitrogen (Camarillo, CA, USA). Antibody for XIAP (2042) was from Cell Signaling (Danvers, MA, USA). Anti-poly (ADP-ribose) polymerase (PARP, ALX-210-222), FLIP (ALX-804-961-0100) were from Enzo Life Sciences (Farmingdale, NY, USA). Antibody for actin (A1978) and LC3B (L7543) was purchased from Sigma-Aldrich (St Louis, MO, USA). Anti-ATG7 (PA5-17216) was from Thermo Scientific (Barrington, IL, USA). The JNK inhibitor SP600125 (420119), Wortmannin (12–338) and MG-132 (474790) were from Calbiochem (La Jolla, CA, USA). Chloroquine (C6628) and 3MA (M9281) were from Sigma-Aldrich. Necrostatin-1 (1864–5) was from BioVision (Milpitas, CA). Pan-caspase inhibitor z-VAD (ALX-260-039) was from Enzo Life Sciences. The ERK inhibitor U0126 (9903) was from Cell Signaling. Short-interfering RNAs for ATG7 (M-020112-01-0005), RIP1 (M-004445-02-0005) and the non-targeting siRNA were purchased from Dharmacon (Lafayette, CO, USA). Chal-24 was synthesized following reported procedures [16]. The FLAG-cIAP1, FLAG-cIAP2 and pEBB-XIAP plasmids were from Addgene (Cambridge MA) [41–43]. The pEGFP-C1 plasmid was from Clontech (Mountain View, CA). The V5-c-FLIP plasmid (HsCD00445121) was purchase from DNASU Plasmid Repository.

### Cell culture

A549, H460, H23 and H1299 cells were obtained from America Type Culture Collection (Manassas, VA, USA) and grown in RPIM 1640 medium supplemented with 10% fetal bovine serum, 2mM L-glutamine, 100 U penicillin and 100 μg/ml streptomycin. All cells were cultured in standard incubator conditions at 37°C with 5% CO_2_.

### Cytotoxicity assay

Cytotoxicity assay was conducted with a cytotoxicity detection kit (Promega) based on the release of lactate dehydrogenase (LDH). Cells were seeded in a 48-well plate at 40–50% confluence. After overnight culture, cells were treated as indicated in each figure legend. LDH release was measured as described previously [44]. Combination index (CI) was calculated as described [45]. To examine the effect of ectopic expression of cIAP2 or c-FLIP on cytotoxicity induced by the Chal-24 and cisplatin combination, A549 cells were transfected 24 h with EGFP and pcDNA, EGFP and c-IAP2 expression plasmids or EGFP and FLIP expression plasmids. EGFP was used as a transfection marker. Then the cells were treated with cisplatin (10 μM) and Chal-24 (1 μM) for 40 h and examined under a fluorescence microscope. The percentage of live cells in the treated samples relative to their respective untreated cells was calculated as described previously [46].

### Western blot and immunoprecipitation

Cell lysates were prepared by suspending cells in M2 buffer (20 mM Tris-HCl pH 7.6, 0.5% NP40, 250 mM NaCl, 3 mM EDTA, 2 mM DTT, 0.5 mM phenylmethylsulfonylfluoride, 20 mM β-glycerophosphate, 1 mM sodium vanadate, and 1 μg/ml leupeptin). Equal amounts of protein from each cell lysates were resolved by 8% or 12% SDS-PAGE and analyzed by Western blot. The proteins were visualized with enhanced chemiluminescence (Millipore) following the instructions of the manufacture. Each experiment was repeated at least three times and representative results are shown. For immunoprecipitation, cells were cultured in 100-mm dishes, treated as indicated in each figure legend, and lysed in M2 buffer. Cell lysates were incubated with 1 mg RIP1 antibody and 20 μl protein A-agarose beads (50%) overnight. Then the beads were washed six times with M2 buffer, and immunoprecipitates were eluted off the beads with electrophoresis sample buffer. The samples were boiled for 5 min and resolved on 12% SDS-PAGE gel. Proteins of interest were detected by Western blot [47, 48].

### Knockdown protein expression by RNAi

A549 and H460 cells were seeded in a 12-well plate and 48-well plate the day before transfection at 40–50% confluence. siRNA was transfected with siRNA INTERFERin (polyplus-transfection.) [48]. Twenty-four hours after transfection, cisplatin (10 μM) and Chal-24 (1 μM) were added to the culture for 48 h and LDH release was measured to examine cisplatin and Chal-24 induced cytotoxicity. Knockdown was confirmed by Western blot.

### Statistical analysis

All data were expressed as mean ± SD and statistical significance was examined with one-way analysis of variance (ANOVA) pairwise comparison. *p* < 0.05 was considered statistically significant.

## SUPPLEMENTARY FIGURES


