# Supplementary material for: Synergistic anticancer effect of cisplatin and Chal-24 combination through IAP and c-FLIPL degradation, Ripoptosome formation and autophagy-mediated apoptosis
Source: Oncotarget. 2015 Feb 12;6(3):1640–51. doi: 10.18632/oncotarget.2746 (PMC4359321; doi:10.18632/oncotarget.2746)
Supplement: Supplementary file 1 [file oncotarget-06-1640-s001.pdf]

SUPPLEMENTARY FIGURES

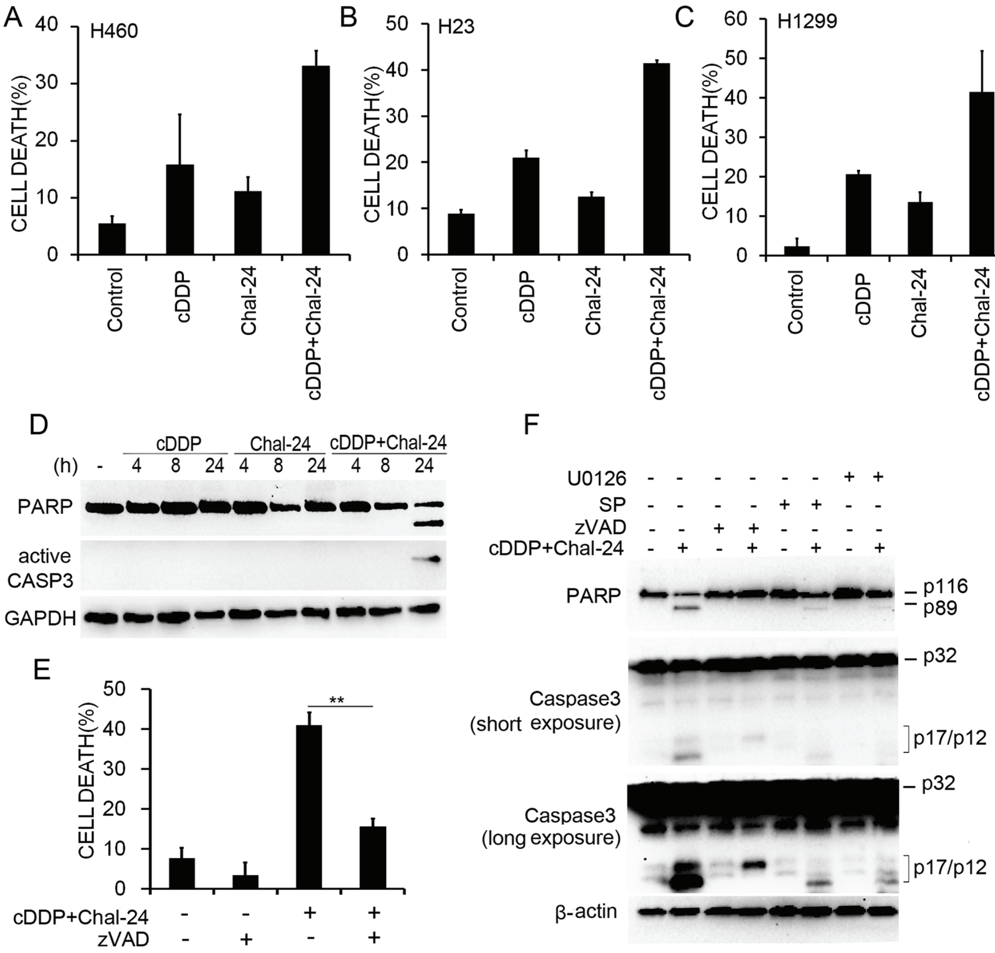

Supplementary Figure S1: Chal-24 and cisplatin co-treatment potentiates apoptosis in human lung cancer cells. (A, B, C) H460, H23 and H1299 cells were treated with cisplatin (5 μM), Chal-24 (1 μM) or in combination for 48 h. Cell death was measured by LDH assay. Data shown are mean ± SD. The data are representative of three independent experiments. **(D)** H460 cells were treated with (5 μM), Chal-24 (1 μM) or in combination for indicated time periods. PARP and active caspase-3 were detected by Western blot. GAPDH was detected as an input control. **(E)** H460 cells were pretreated with z-VAD-fmk (10 μM) for 30 min, followed by 48 h treatment with cisplatin (5 μM) and Chal-24 (1 μM), cell death was detected by LDH assay. ** $p < 0.01$. **(F)** A549 cells were pretreated with z-VAD-fmk (10 μM), SP600125 (10 μM), U0126 (10 μM) for 30 min, then treated with Chal-24 (1.0 μM) and cisplatin (10 μM) for additional 24 h, PARP and caspase-3 were detected by Western blot. β-actin was detected as an input control.

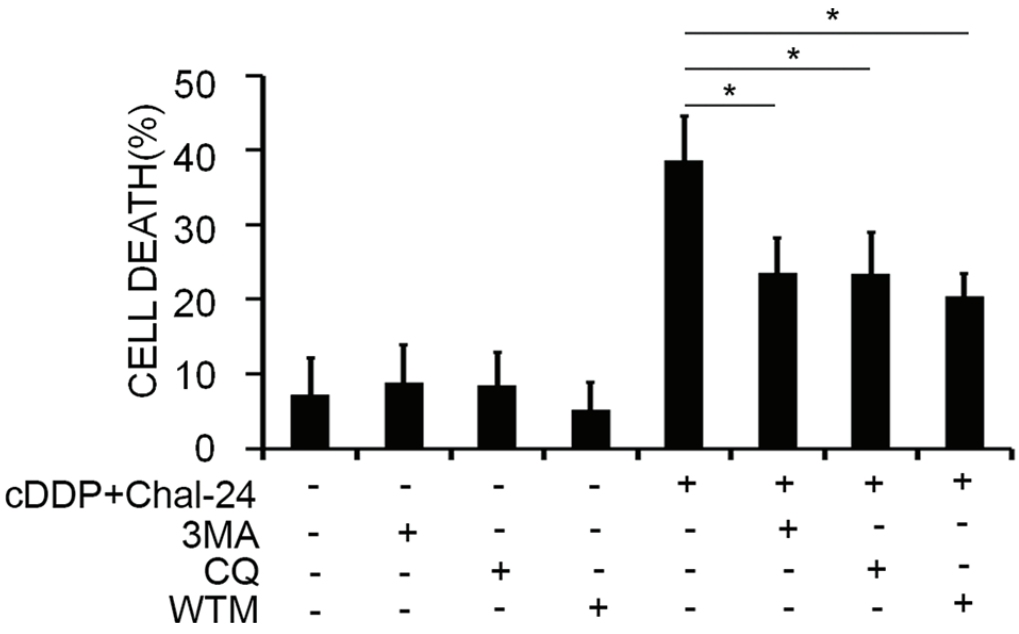

Supplementary Figure S2: Chal-24 and cisplatin co-treatment induces cell death depending on autophagy. H460 cells were pretreated with (CQ, 20 µM; WTM, 1 µM; 3MA, 10 µM) for 30 min, followed by cisplatin (5 µM) and Chal-24 (1 µM) co-treatment for additional 48 h, cell death was measured by LDH assay. *$p < 0.05$.

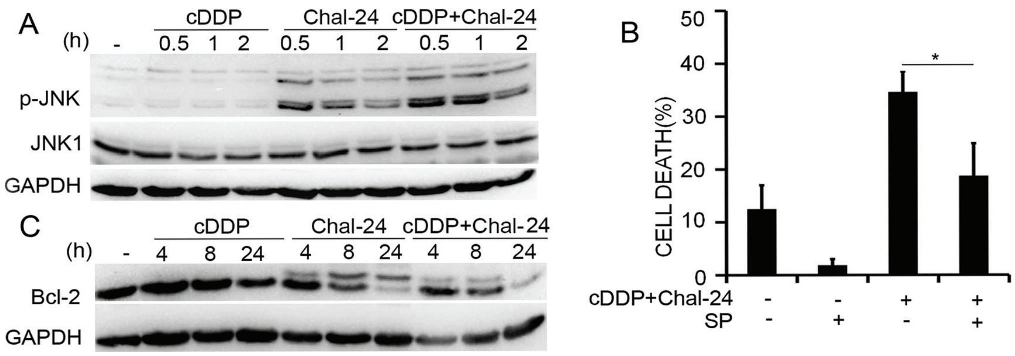

Supplementary Figure S3: Chal-24 and cisplatin co-treatment activates JNK activation and Bcl-2 phosphorylation. (A) H460 cells were treated with cisplatin (5 µM), Chal-24 (1 µM), or in combination for indicated times. JNK1 and phosphor-JNK were examined by Western blot. GAPDH was detected as an input control. **(B)** the cells were pretreated with SP600125 (10 µM) for 30 min, and then treated with cisplatin (5 µM) and Chal-24 (1 µM) for an additional 48 h. Cell death was detected by LDH assay. **(C)** H460 cells were treated with cisplatin (5 µM), Chal-24 (1 µM), or in combination for indicated times. Bcl-2 was examined by Western blot. GAPDH was detected as an input control. *$p < 0.05$.

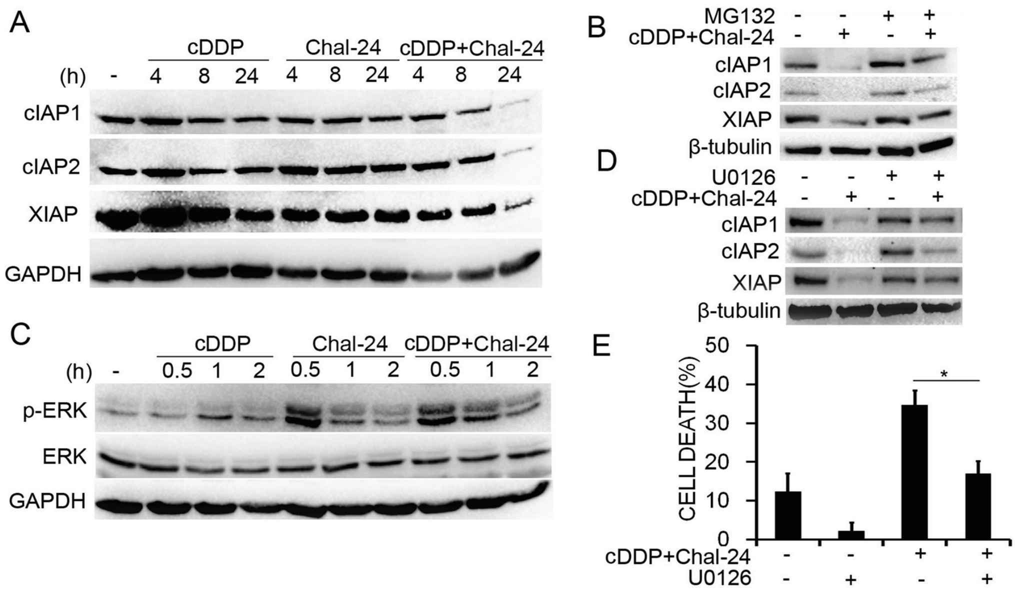

Supplementary Figure S4: Chal-24 and cisplatin co-treatment induces proteasomal degradation of IAP proteins. (A) H460 cells were treated with cisplatin (5 µM), Chal-24 (1 µM), or in combination for indicated times. The indicated proteins were examined by Western blot. GAPDH was detected as an input control. **(B)** the cells were pretreated with MG132 (5 µM) for 30 min, and then treated with cisplatin (5 µM) and Chal-24 (1 µM) for an additional 24 h, the indicated proteins were detected by Western blot. β-tubulin was detected as an input control. **(C)** H460 cells were treated with cisplatin, Chal-24, or in combination for indicated times. The indicated proteins were examined by Western blot. GAPDH was detected as an input control. **(D)** the cells were pretreated with U0126 (10 µM) for 30 min, and then treated with cisplatin (5 µM) and Chal-24 (1 µM) for an additional 24 h, the indicated proteins were detected by Western blot, β-tubulin was used as an input control. **(E)** the cells were pretreated with U0126 (10 µM) for 30 min, and then treated with cisplatin (5 µM) and Chal-24 (1 µM) for an additional 48 h. Cell death was detected by LDH assay. *$p < 0.05$.

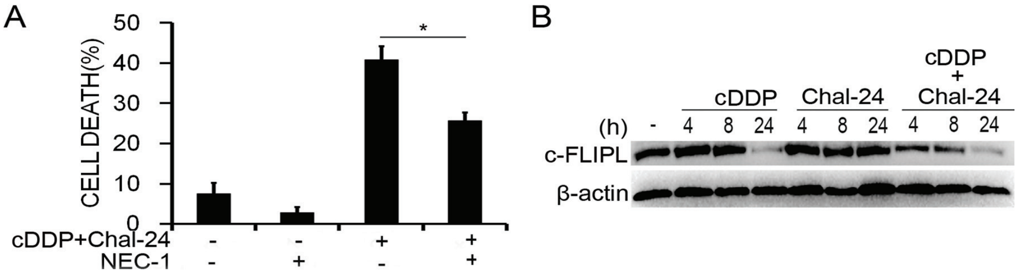

Supplementary Figure S5: Cell death induced by Chal-24 and cisplatin co-treatment involves RIP1 activity and the co-treatment induces cFLIP degradation. (A) H460 cells were pretreated with necrostatin-1 (NEC-1, 10 µM) for 30 min, and then treated with cisplatin (5 µM) and Chal-24 (1 µM) for an additional 48 h. Cell death was measured by LDH assay. **(B)** H460 cells were treated with cisplatin, Chal-24, or in combination for indicated times. The indicated proteins were examined by Western blot. β-actin was detected as an input control. *$p < 0.05$.
